# The efficacy of a clustered group-based acceptance and commitment therapy for patients with chronic pain — a randomized controlled semi-crossover trial

**DOI:** 10.1080/24740527.2025.2515106

**Published:** 2025-07-31

**Authors:** Lena Danielsson, Svein Bergvik, Are Hugo Pripp, Gunnvald Kvarstein

**Affiliations:** a Department of Pain Management, Division of Surgical Medicine and Intensive Care, University Hospital of North Norway, Tromsø, Norway; b Department of Clinical Medicine, UiT - the Arctic University of Norway, Tromsø, Norway; c Department of Psychology, UiT the Arctic University of Norway, Tromsø, Norway; d Oslo Centre of Biostatistics and Epidemiology, Research Support Services, Oslo University Hospital, Oslo, Norway; e Faculty of Health Sciences, OsloMet – Oslo Metropolitan University, Oslo, Norway; f Department of Pain Management and Research, Division of Emergencies and Critical Care, Oslo University Hospital, Oslo, Norway

**Keywords:** Acceptance and commitment therapy, chronic pain, group intervention, clustered intervention, randomized control trial, Semi-crossover design

## Abstract

**Purpose:**

The efficacy of Acceptance and Commitment Therapy (ACT) for chronic pain when provided as weekly sessions, is well documented. In scarcely populated areas, the traveling distance may be a barrier to weekly attendance. This study aimed to test the efficacy of a group-based ACT intervention, clustered into three bouts of three consecutive days, separated by 4 weeks.

**Patients and methods:**

A total of 122 patients, recruited from a university hospital pain clinic, were randomized to either a clustered ACT or Treatment As Usual (TAU) provided by the primary health care services. The study had a semi-crossover design. Group effects of ACT versus TAU were assessed 3 months after the start of ACT by using linear mixed models for repeated measures. Outcome measures included pain intensity, health-related quality of life, pain acceptance, catastrophizing, and psychological distress.

**Results:**

A total of 81 patients completed the ACT intervention. No statistically significant effects were observed on the primary outcome variables, pain intensity and health-related quality of life.

Significant group differences in favor of ACT were detected in pain acceptance (modified Cohen`s d = 0.32), including pain willingness (modified Cohen`s d = 0.30) and activity engagement (modified Cohen`s d = 0.23). The treatment effect remained at the 6- and 12-month follow-ups with a trend toward improvement.

**Conclusion:**

A group-based ACT for chronic pain clustered into 3-day bouts may strengthen pain acceptance processes, including pain willingness and activity engagement. Reasons why the intervention did not affect pain intensity and health-related quality of life are discussed.

## RÉSUMÉ

**Objectif:** L’efficacité de la thérapie d’acceptation et d’engagement dans le traitement de la douleur chronique, lorsqu’elle est offerte sous forme de séances hebdomadaires, est bien documentée. Toutefois, dans les régions peu peuplées, la distance à parcourir peut constituer un obstacle à la participation hebdomadaire. Cette étude visait à évaluer l’efficacité d’une intervention de groupe fondée sur la thérapie d’acceptation et d’engagement, structurée en trois blocs de trois jours consécutifs, espacés de quatre semaines.

**Patients et méthodes:** Un total de 122 patients recrutés dans une clinique de la douleur d’un hôpital universitaire a été réparti aléatoirement dans un groupe recevant la thérapie d’acceptation et d’engagement ou dans un groupe recevant les soins habituels fournis dans le cadre des soins de santé primaires. L’étude reposait sur un devis semi-croisé. Les effets de la thérapie d’acceptation et d’engagement comparativement aux soins habituels ont été évalués trois mois après le début de l’intervention, à l’aide de modèles linéaires mixtes pour mesures répétées. Les mesures des résultats comprenaient l’intensité de la douleur, la qualité de vie liée à la santé, l’acceptation de la douleur, ainsi que la catastrophisation et la détresse psychologique.

**Résultats:** Au total, 81 patients ont complété l’intervention fondée sur la thérapie d’acceptation et d’engagement. Aucune différence statistiquement significative n’a été observée pour les variables de résultats principales, soit l’intensité de la douleur et la qualité de vie liée à la santé. En revanche, des différences significatives entre les groupes, en faveur de la thérapie d’acceptation et d’engagement, ont été observées pour l’acceptation de la douleur (d de Cohen modifié = 0,32), y compris pour la disposition à éprouver la douleur (d de Cohen modifié = 0,30) et l’engagement dans les activités (d de Cohen modifié = 0,23). L’effet du traitement s’est maintenu lors des suivis à 6 mois et à 12 mois, avec une tendance à l’amélioration.

**Conclusion:** Une intervention de groupe fondée sur la thérapie d’acceptation et d’engagement, structurée en blocs de trois jours pour le traitement de la douleur chronique, peut renforcer les processus d’acceptation de la douleur, notamment la disposition à éprouver la douleur et l’engagement dans les activités. Les raisons pour lesquelles l’intervention n’a pas eu d‘effet sur l’intensité de la douleur ni sur la santé ou la qualité de vie sont discutées.

## Introduction

Pain, defined as *“an unpleasant sensory and emotional experience associated with, or resembling that associated with, actual or potential tissue damage*”^1^ is a significant physiological sign of bodily threat, prompting protective behaviors such as avoidance, withdrawal, and vigilant attention to the source of pain.^2^ While these behaviors act as adaptive and pertinent responses to acute injury, they are ineffective and may even worsen and perpetuate chronic pain, reducing physical function and quality of life.^3–5^

Biased cognitions such as catastrophizing thoughts are prevalent in patients with chronic pain and refer to a negative “cognitive set” and overestimation of the probability of unpleasant outcomes.^6^ Pain catastrophizing is further associated with avoidant behavior,^7–9^ helplessness, rumination, and negative attentional focus,^10^ and is a well-known predictor of pain.^11–14^

Evidence-based psychological approaches are now broadly implemented in the treatment of chronic pain.^15^ Acceptance and Commitment Therapy (ACT) represents a third-generation cognitive therapy based on the Relational Frame Theory (RFT).^16–18^ The RFT is described as a contemporary behavior analytic approach, aiming to improve understanding of the link between human language and behavior.^18^

ACT aims to build psychological flexibility and thereby improve the ability to act in accordance with motivating values and goals, even in the presence of health issues such as pain with the associated cognitions and emotions. ACT is guided by six therapeutic processes toward psychological flexibility: *acceptance, committed action, values, defusion, contact with the present moment*, and *self-as-context*. These processes have been thoroughly described by Hayes, Strosahl, and Wilson.^19,20^ ACT employs various techniques to strengthen psychological flexibility, including pain acceptance, and includes strategies to identify personal values and goals, mindfulness and experiential exercises, behavioral modification techniques, and the use of metaphors to raise awareness of and assist in overcoming the dominating role of verbal behavior.^21^

ACT has gained increased popularity as a potentially cost-effective treatment for chronic pain.^22,23^ A systematic review and meta-analysis of ACT for chronic pain demonstrated low-to-moderate evidence for short-term effects on physical, social, and general functioning. However, the findings for long-term effects were less consistent. They found moderate evidence for sustained effects in fibromyalgia patients, but not for patients with mixed chronic pain or nonspecific low back pain.^24^

Randomized ACT trials are typically based on 8–12 weekly group sessions, each of a 60 to 120 minutes duration.^24–27^ Frequent sessions, however, require that patients reside reasonably close to the treatment center. Thus, in scarcely populated areas with long traveling distances, weekly treatment sessions are not feasible for many patients.^28^ Internet-delivered ACT is an alternative, but in studies, this format has been hampered by poor program adherence and high attrition rates.^29–34^

The current clinical trial took place at a university hospital that serves patients from a wide geographical area, with some residing up to 790 km away. Many patients travel considerable distances by car or public transportation, while others need to use air transport to reach the facility. Clinical observations indicate that many patients attending the Pain Department experience significant fatigue due to long and demanding trips to the hospital. Some require weeks to recover from the strain of such a travel.

To alleviate the burden of repetitive travels and thereby improve accessibility, a clustered intervention was developed. The intervention consisted of nine days of group therapy, delivered in three bouts with four-week intervals. This clustered scheduling allowed sufficient time for recovery and engagement with home-based exercises between sessions.

This model offers an alternative to conventional weekly face-to-face or internet-based interventions. To our knowledge, this is the first study to implement a clustered group format of Acceptance and Commitment Therapy (ACT) for patients with chronic pain.

The trial aimed to assess the efficacy of a clustered, group-based ACT intervention compared to treatment-as-usual (TAU) provided by the primary health care services. The primary hypothesis was that a clustered group-based ACT treatment leads to less pain and greater Health Related Quality of Life (HRQOL) in patients with chronic pain compared with treatment in a primary care setting. Additionally, it was expected that secondary outcomes, including pain acceptance (activity engagement and pain willingness), psychological distress, and pain catastrophizing, would show significant improvement in participants receiving ACT compared to those receiving TAU.

## Materials and methods

### Design

The trial compared a group-based ACT intervention at the University Hospital against TAU provided by the primary healthcare services. A randomized controlled, semi-crossover design was used to enhance recruitment by ensuring that all participants were offered ACT and to increase the size of the intervention group. Participants were equally randomized to either an “early” ACT group or a TAU group. The latter group were later offered ACT (“late” ACT). Those assigned to TAU (“late” ACT”) served both as the control group (TAU) and the interventional group (“late” ACT) in the analysis. The only group comparison was conducted after 3 months.

The trial adhered to the CONSORT 2010 checklist for randomized crossover trials for social and psychological interventions, including a noncontrolled 6- and 12-month extension.^35–37^

### Recruitment

We recruited patients with various forms of chronic noncancer pain referred to an outpatient pain department at a university hospital between 2016 and 2020. All patients were subjected to an interdisciplinary assessment by a team consisting of a pain physician, a psychologist or psychiatric nurse, and a physiotherapist. Patients considered eligible for the group intervention were offered a second evaluation by a trained ACT therapist, who provided information about the trial and invited them to participate.

Eligibility criteria were individuals aged 18 years or older, a pain duration of more than 6 months, and motivation to participate in the group-based ACT trial. Patients with a severe psychiatric or progressive disorder, drug addiction, or consumption of >100 oral morphine equivalents/day were excluded.

### Randomization

**Figure 1.**
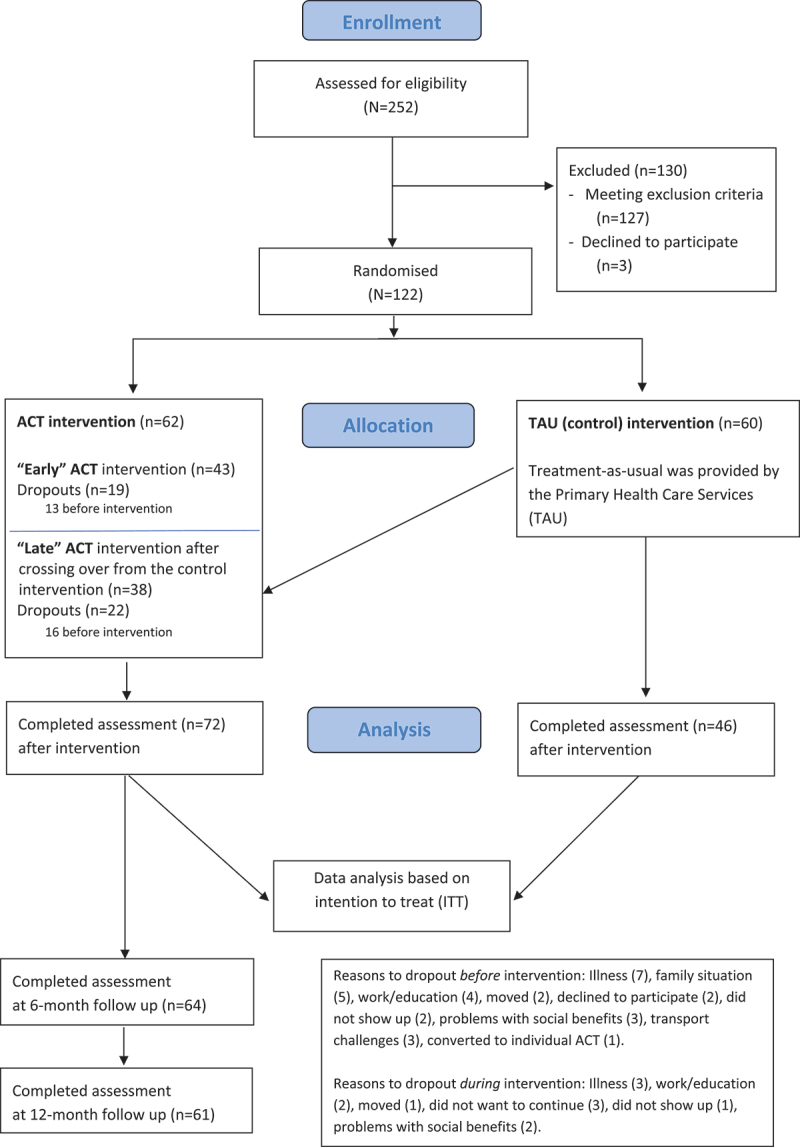
Flow chart.

Participants were randomized to either “early” ACT or to TAU provided by the primary health care services. TAU participants crossed over to receive “late” ACT 3 months later (Figure 1). A computer-based, gender-stratified block randomization was developed and administered by the Clinical Research Department at the hospital by personnel not involved in the clinical evaluation or the ACT intervention. Each block included three participants. Due to the consecutive recruitment and varying time spans between randomization and the ACT intervention, both patients and clinicians remained unaware of their group allocation.

### ACT intervention

The ACT intervention included a total of nine days of group sessions, clustered into bouts of 3 consecutive days, each bout separated by 4 weeks. To be considered an intervention-completer, participants were required to attend at least two of the three 3-day-bouts (≥6 days in total), aligning with prior studies.^38–40^ Mean group size was 7.6 (*SD* 1.7). The out-of-town participants were housed near the treatment center.

A pool of seven trained ACT therapists facilitated the sessions, working in alternating pairs. The therapists represented experienced clinicians from diverse professional backgrounds – psychologists, physiotherapists, and a psychiatric nurse – all trained in ACT and employed at the pain department. Additionally, the therapists received monthly supervision from an external ACT therapist with extensive experience in the field.

The intervention followed a detailed treatment manual, addressing topics within the major concepts of ACT. The participants received a written information folder on the first day of treatment. Each bout had daily 4.5–5 hours sessions, including breaks. The treatment combined short lectures with group exercises and discussions, including mindfulness exercises. Each 3-day bout focused on an overall theme with a subtheme for each respective day (Table 1). The participants were instructed to continue working at home on chosen exercises and tasks until the next bout. During this 4-week interval, they received an individual telephone consultation by one of the ACT therapists, providing guidance and encouragement. Three months after completing the intervention, all participants met individually with one of the ACT therapists for a face-to-face consultation. The participants were not financially compensated but received coverage for transportation, accommodation, and meals.

**Table 1.** Main themes of the Acceptance and Commitment Therapy (ACT) intervention.

First bout	Day 1	Introducing ACT through the “lifeline,” a tool that helps visualize the life journey and evaluate how actions align with values. Exercises in creative hopelessness to demonstrate that past attempts to control or avoid suffering have been ineffective, creating space for new, values-driven actions. Mindfulness: The practice of being fully present and aware of your thoughts, feelings, and surroundings without judgment.
	Day 2	Pain physiology. Exercises in creative hopelessness, example: the “Struggle Tug-of-War” metaphor. Values vs. goals: Values provide meaning, while goals are the steps taken to live according to those values. Mindfulness exercises.
	Day 3	Mindfulness exercises. Values: The “Bull’s Eye” exercise helps to assess how closely actions align with core values across key life areas (e.g., relationships, work, health). Committed actions: Exercises to build consistency in living according to values through meaningful actions.
Second bout	Day 1	Values exercises. Defusion: Exercises to help create distance from thoughts, making them less overwhelming. Self-as-context is the idea that you are not your thoughts, emotions, or experiences – you are the observer of them. Example: Imagine thoughts as clouds in the sky. You are not the clouds; you are the sky, watching them come and go. Acceptance: Allowing thoughts, emotions, and sensations to be present without trying to fight, avoid, or control them. Mindfulness exercises.
	Day 2	Defusion and Acceptance, example: “Leaves on a Stream” exercise to accept thoughts and emotions while defusing from them, allowing them to come and go without controlling you. Mindfulness exercises.
	Day 3	Acceptance and Willingness to change, example: The “Kindness Meditation” encourages embracing emotions (even difficult ones) and cultivating kindness toward oneself and others. Mindfulness exercises.
Third bout	Day 1	Experiential avoidance, example: “Demons on the boat” Metaphor: You’re steering a boat toward your values when demons (fears, doubts) appear, threatening you. Fighting them stops your progress. Instead, let them stay on the boat and keep moving forward. Mindfulness exercises.
	Day 2	Experiential avoidance. Discussion about preventing relapse. Mindfulness exercises.
	Day 3	The ACT Matrix a tool for increasing psychological flexibility by organizing experiences into four quadrants: Away Moves (actions driven by avoidance, like procrastination or withdrawal), Toward Moves (actions aligned with values, such as connecting with loved ones or pursuing goals), Internal Experiences (thoughts, emotions, and sensations like fear or self-doubt), and External Actions (observable behaviors, such as speaking up or exercising). The end and the way forward. Summary: The ACT Hexaflex with the six core processes of psychological flexibility. Mindfulness exercises.

### Treatment as Usual (TAU)

Patients in the control group (TAU) received care from the primary care services, primarily provided by the general practitioner (GP). Both the GP and patient received a discharge note, which included diagnoses and a multidisciplinary evaluation of the pain condition. No new treatments were introduced during the control period. The participants, representing a heterogeneous group with chronic pain, may have received various treatments by their GP, in both content and format. However, we have no detailed data on these treatments.

### Data collection

All participants completed online questionnaires at baseline, just before and after the intervention (ACT and TAU), and at the 6- and 12-month follow-ups.

### Socio-demographic variables

Socio-demographic data included gender, age, total years of education (dichotomized into ≤ 12 or >12 years), working/in education status (“work status”) (dichotomized to yes = full or part-time work or study, no = no work or study), and their perceived financial situation (dichotomized into poor vs. medium/good).

### Primary outcome measures

#### Pain intensity

The participants reported their most severe, least severe, and average pain intensity during the last week, as well as their current pain intensity at the time they completed the questionnaires. The intensity was graded on an 11-point Numeric Rating Scale (NRS) ranging from 0 = no pain to 10 = worst possible pain.^41^ The four pain scales have been applied in the Brief Pain Inventory (BPI), and validation has shown satisfactory properties in a Norwegian sample.^42,43^ In this trial, a composite pain intensity score was calculated as the average of the four pain assessment scores.

#### Health-related quality of life

The Short form-36 version 2 (SF-36v2) is a self-report questionnaire assessing Health-related Quality of Life. The 36 items assess the following eight health domains: 1) limitations in physical activities due to health problems, 2) limitations in social activities due to physical or emotional problems, 3) limitations in usual role activities due to physical health problems, 4) bodily pain, 5) general mental health (psychological distress and well-being), 6) limitations in usual role activities due to emotional problems, 7) vitality (energy and fatigue), and 8) perceived general health.^44^ Higher scores indicate better health-related quality of life. The internal consistency and construct validity of the Norwegian version have been proved acceptable for all scales, and across all scales, with Cronbach’s alpha coefficients surpassing the 0.70 threshold.^45^ In a low back pain population, Cronbach’s alpha coefficients for all eight individual scales ranged from 0.72 to 0.93.^46^ Norm-based scores are recommended,^47^ and correlations between the SF-36 summary measures scores, using standard (U.S.) scoring algorithms and country-specific scoring algorithms, have been shown very high.^48^ If a health domain scale or component summary measure falls outside the T-score range of 45 to 55 it should be considered outside the average range.^47^

### Process variables and secondary outcome measures

#### Chronic Pain Acceptance Questionnaire-Revised (CPAQ-R)

The 20-item version of CPAQ-R^49^ is a self-report measure of pain acceptance. Each item has a 7-point response format ranging from 0 (never true) to 6 (always true). A CPAQ-R total score and the two subscores activity engagement (Items 1, 2, 3, 5, 6, 8, 9, 10, 12, 15, 19) and pain willingness (Items 4, 7, 11, 13, 14, 16, 17, 18, 20) are calculated by summarizing the respective items.^49^ High scores indicate high levels of pain acceptance. The CPAQ-R has demonstrated good internal consistency, with alphas of .82 (activity engagement) and .78 (pain willingness), respectively, and moderate-to-high correlations with measures of distress, avoidance, and daily functioning. The two factors of the CPAQ-R are found to significantly predict pain-related disability and distress, demonstrating predictive validity.^50,51^

#### Pain catastrophizing

The Pain Catastrophizing Scale (PCS) is a 13-item self-report measure of catastrophic thinking related to pain in adult populations with chronic pain.^52^ The participants are asked to consider past painful experiences and rate each item in relation to their pain experience on a 5-point scale where 0 = not at all and 4 = all the time. The sum score ranges from 0 to 52, where high scores indicate high levels of catastrophizing. PCS includes three subscales, assessing rumination (items 8, 9, 10, 11, 2), magnification (items 6, 7, 13), and helplessness (items 1, 2, 3, 4, 5, 12), respectively.^53,54^ The PCS has demonstrated adequate to excellent internal consistency (alphas: total PCS =.87, rumination =.87, magnification =.66, and helplessness =.78.^52,55^

#### Psychological distress

The Hopkins Symptom Checklist-25 (HSCL-25) is a widely used, self-administrated measure of symptoms of depression and anxiety. Each of the 25 items has a four-point response format ranging from 1 = not at all to 4 = extremely. The average item score is calculated by dividing the total sum score by the number of items answered.^56,57^ It is considered a valid and reliable measure of psychological distress.^58^ A cutoff of 1.75 for women and 1.67 for men has been suggested as a valid predictor of psychological distress.^57–59^

#### Fatigue

The Chalder Fatigue Questionnaire (CFQ) is a self-report instrument designed to measure the severity of physical and mental fatigue. It was developed to assess fatigue in both clinical and nonclinical populations.^60^ The CFQ consists of 11 items divided into the two subscales physical fatigue and mental fatigue. Respondents rate each item on a four-point Likert scale ranging from 0 = less than usual to 3 = much more than usual, providing total sum score (range 0–33). The CFQ has demonstrated good reliability and validity.^61^

### Assessment of adverse events

The therapists asked the patients during the sessions and in the face-to-face consultation 3 months after the ACT intervention about potential adverse events.

### Statistical methods

Statistical analyses were performed using IBM SPSS Statistics for Windows, version 28.0 (IBM Corp, Armonk, NY, USA) and Stata/SE 17.0 for Windows (StataCorp LLC, College Station, TX, USA). Continuous data are reported as means with standard deviation (SD), and categorical data as numbers and percentages of observations, respectively. The statistical distributions were checked with box plots. We assessed the semi-crossover design using both full and restricted linear mixed models for repeated measurements with a subject-specific intercept. The full model contained fixed factors for group (i.e., “early” ACT, “late” ACT and control (TAU) intervention), time (i.e., before intervention, after 3-month intervention, 6-month follow-up and 12-month follow-up), interaction between fixed effects of group and time, and the outcome measures at baseline as a covariate. Given the crossover of TAU participants into “late” ACT after 3 months, the data from the 6- and 12-month follow-ups were based solely on the “early” and “late” ACT groups. Then, we estimated a restricted (nested) model with “early” and “late” ACT combined into one ACT group with thereby fixed factors for group (i.e. ACT and control (TAU) intervention), time (i.e. before intervention, after 3-month intervention, 6-month follow-up and 12-month follow-up), interaction between fixed effects of group and time, and the outcome measures at baseline as a covariate. As in the full model, the restricted model only had data from the ACT group for the 6- and 12-month follow-ups due to the cross-over.

To assess potential period or carryover effects in the semi-crossover design (systematic difference between “early” and “late” ACT), we estimated a likelihood-ratio test between the full and restricted linear mixed model. With a nonsignificant likelihood-ratio test, we rejected the hypothesis of period or carryover effects and reported only results from the restricted model.

Treatment effects are presented as means and mean group differences with standard error of the mean (SEM) and 95% confidence intervals (CI) estimated from the linear mixed models. We calculated a modified Cohen’s “d” as the mean group difference divided by the standard deviation at baseline (Table 3).

Due to the extended time gap between randomization and the intervention, we used pre-intervention data in the statistical analyses in order to provide more reliable estimates of the intervention’s effects.^62,63^ The analyses also adjusted for the outcome measures at baseline.

Statistical analyses were performed on an intention-to-treat (ITT) basis. To account for participant dropout, secondary per-protocol (PP) analyses were conducted. LMMs manage missing data by using Maximum Likelihood (ML) estimation, which provides unbiased estimates under the assumption that data is Missing at Random (MAR), thus reducing potential bias compared to traditional methods such as complete case analysis.^64^

### Sample size

Based on RCTs published prior to the onset of our trial, we found an effect size of 0.55 to be relevant.^65^ Given a statistical power of 80%, a significance level of 5%, and a semi-crossover design providing an approximated group ratio of 2:1, this required a study sample of 78 participants (i.e., all 78 participants in the treatment arm and 39 of them also in the control arm). We assumed a low within-subject correlation and did not consider it in this sample size calculation. Considering the risk of high dropout rates and the number of patients who received ACT in our department, we included 120 patients.

## Results

### Attendance

A total of 122 participants were included and randomly allocated to “early” ACT intervention (*n* = 62) or the TAU administered by the primary healthcare service (*n* = 60) before crossing over to “late” ACT intervention.

Forty-one participants dropped out of the intervention. Twenty-nine of these dropped out before the onset of ACT and 12 during the ACT program. Thus, a total of 81 participants completed the ACT intervention. For details on attendance, drop-outs, and questionnaire completion see Figure 1 (Flow chart).

### Participants

Table 2 delineates the sociodemographic and outcome measures collected at baseline. The initial assessment revealed no significant disparities between the groups, indicating a successful randomization of the study sample. In line with the CONSORT guidelines (Moher et al., 2010), we compared the two randomized groups based on clinical judgment rather than statistical testing for group differences.^66^

**Table 2.** Baseline group comparisons of sociodemographic and clinical characteristics in the study sample of chronic pain patients.

		ACT		TAU (control)		Dropouts
	*N*	*mean (SD) or number (%)*	*n*	*mean (SD) or number (%)*	*n*	*mean (SD) or number (%)*
Age (years)	122	45.9 (*SD* 10.8)	60	45.0 (*SD* 10.0)	41	42,5 (*SD* 10,4)
Gender (females)	122	96 (79%)	60	48 (80%)	41	31 (75%)
Having children	116	96 (82%)	57	49 (86%)	36	30 (83%)
Habitational status (living alone)	117	17 (14%)	59	11 (18%)	36	7 (19%)
Length of education	117		59		36	
<10 years		16 (14%)		4 (7%)		4 (11%)
10–12 years		54 (46%)		27 (46%)		20 (56%)
12 years +		47 (40%)		28 (47%)		12 (33%)
Occupational status	116		58		36	
Working		33 (28%)		21 (36%)		12 (33%)
Student		2 (2%)		1 (2%)		0 (0%)
Not working		81 (70%)		36 (62%)		24 (67%)
Percieved financial situation	115		57		36	
Good		26 (23%)		14 (25%)		9 (25%)
Medium		63 (54%)		29 (50%)		17 (47%)
Poor		26 (23%)		14 (25%)		10 (28%)
Pain duration	107		54		32	
1.5–9 years		53 (50%)		22 (41%)		17 (53%)
10–20 years		39 (36%)		24 (44%)		9 (28%)
>20 years		15 (14%)		8 (15%)		6 (19%)
Duration of current pain level	117		59		35	
<1 year		12 (10%)		5 (8%)		3 (9%)
≥1 year		105 (90%)		54 (92%)		32 (91%)
Widespread pain	116	60 (51%)	59	26 (44%)	36	16 (44%)
Pain intensity (NRS)*	115	5,4 (*SD* 1,6)	58	5,1 (*SD* 1,3)	36	5,5 (*SD* 1,6)
Quality of life (SF-36v2) domains:						
Physical Functioning	116	40,5 (*SD* 8,8)	58	41,8 (*SD* 8,1)	35	41,3 (*SD* 9,1)
Role-Physical	116	31,5 (*SD* 9,8)	58	31,0 (*SD* 9,8)	35	31,6 (*SD* 9,2)
Bodily Pain	115	31,6 (*SD* 6,6)	57	32,6 (*SD* 7,1)	35	32,1 (*SD* 5,0)
General Health	116	36,0 (*SD* 9,9)	58	35,6 (*SD* 9,9)	35	37,1 (*SD* 9,4)
Vitality	116	33,2 (*SD* 8,4)	58	33,3 (*SD* 8,2)	35	31,1 (*SD* 5,7)
Social Functioning	116	33,3 (*SD* 11,1)	58	32,4 (*SD* 10,5)	35	33,6 (*SD* 9,7)
Role-Emotional	113	43,7 (*SD* 13,0)	55	46,7 (*SD* 11,1)	35	42,9 (*SD* 14,8)
Mental Health	116	45,1 (*SD* 9,6)	58	45,7 (*SD* 9,7)	35	45,3 (*SD* 8,4)
Psychological distress (HSCL-25)	109	2,0 (*SD* 0,5)	52	1,9 (*SD* 0,5)	32	2,1 (*SD* 0,5)
Pain catastrophizing (PCS)	113	20,2 (*SD* 11,0)	55	19,5 (*SD* 10,3)	34	20,3 (*SD* 11,0)
Pain acceptance total (CPAQ-R)	111	53,2 (*SD* 16,3)	55	52,9 (*SD* 15,9)	34	52,7 (*SD* 15,9)
Pain willingness (CPAQ-R)	111	21,4 (*SD* 8,2)	55	21,4 (*SD* 7,9)	34	21,0 (*SD* 9,0)
Activity engagement (CPAQ-R)	112	31,5 (*SD* 11,6)	55	31,5 (*SD* 11,2)	34	31,7 (*SD* 9,5)
Fatigue (CFQ)	113	20,5 (*SD* 5,8)	55	20,5 (*SD* 4,9)	32	21,6 (*SD* 5,4)

Note: ACT = Acceptance and Commitment Therapy at a multidisciplinary pain clinic; TAU = Treatment-As-Usual provided by the primary health care services; SF-36v2 = Short Form survey 36 version 2; NRS = Numeric Rating Scale; HSCL-25 = Hopkins Symptom Check List-25; PCS = Pain Catastrophizing Scale; CPAQ-R = Chronic Pain Acceptance Questionnaire - Revised; CFQ = Chalder Fatigue Questionnaire; SD = Standard Deviation. *Pain Intensity is the mean value of strongest, weakest, mean and current pain intensity.

### Post-treatment outcome results

#### Primary outcomes

The linear mixed models did not detect any significant group differences between the ACT and TAU on the primary outcome measures, neither for pain intensity nor health-related quality of life (Table 3) although approximately half of the participants reported improvement on pain intensity and health-related quality of life; see Table 4. We further observed a nonsignificant trend, favoring ACT for social functioning (a subscale of the health-related quality of life measure).

**Table 3.** Group comparisons of patients with chronic pain 3 months after onset of Acceptance and Commitment Treatment versus Treatment-As-Usual and follow-up data at the 6- and 12-month follow-ups.

Variables	Group	Before intervention *mean (SEM)*	After 3-month intervention *mean (SEM)*	Group differences after 3-month intervention *mean (CI 95%)*	Effect size *Modified Cohen’s d ^$^*	6-month follow-up *mean (SEM)*	12-month follow-up *mean (SEM)*
Quality of life (SF-36v2) domains:							
Physical Functioning	ACT	41,5 (0,60)	42,2 (0,66)	−0,5 (−2,2 to 1,2)	0,06	41,5 (0,69)	41,0 (0,70)
	TAU	42,6 (0,75)	41,7 (0,81)				
Role-Physical	ACT	32,4 (0,79)	32,5 (0,87)	−0,4 (−2,8 to 1,9)	0,05	31,8 (0,91)	34,1 (0,93)
	TAU	32,8 (1,00)	32,0 (1,08)				
Bodily Pain	ACT	32,2 (0,53)	32,7 (0,60)	0,6 (−1,1 to 2,4)	−0,09	32,1 (0,64)	33,2 (0,65)
	TAU	33,0 (0,69)	33,3 (0,76)				
General Health	ACT	36,7 (0,68)	36,8 (0,75)	0,4 (−1,5 to 2,3)	−0,04	35,3 (0,79)	36,2 (0,79)
	TAU	37,2 (0,85)	37,1 (0,91)				
Vitality	ACT	33,8 (0,76)	35,4 (0,84)	−1,2 (−3,4 to 0,9)	0,15	34,1 (0,89)	34,9 (0,90)
	TAU	33,1 (0,95)	34,1 (1,03)				
Social Functioning	ACT	33,3 (0,97)	35,8 (1,10)	−2,4 (−5,4 to 0,6)	0,22	35,0 (1,15)	35,9 (1,17)
	TAU	32,1 (1,24)	33,4 (1,37)				
Role-Emotional	ACT	41,7 (1,21)	43,0 (1,35)	−0,3 (−4,1 to 3,5)	0,02	40,2 (1,430)	43,6 (1,45)
	TAU	42,1 (1,56)	42,7 (1,73)				
Mental Health	ACT	46,0 (0,80)	46,2 (0,88)	1,2 (−1,0 to 3,4)	−0,13	44,9 (0,93)	47,3 (0,93)
	TAU	44,8 (0,99)	47,4 (1,06)				
Pain intensity (NRS)	ACT	5,2 (0,13)	4,9 (0,14)	0,1 (−0,3 to 0,4)	−0,03	5,0 (0,15)	5,0 (0,15)
	TAU	5,3 (0,16)	5,0 (0,17)				
Psychological distress (HSCL-25)	ACT	1,9 (0,03)	1,9 (0,03)	0,0 (−0,1 to 0,1)	−0,02	1,9 (0,03)	1,9 (0,04)
	TAU	2,0 (0,04)	1,9 (0,04)				
Pain catastrophising (PCS)	ACT	17,9 (0,80)	16,6 (0,88)	−0,5 (−2,6 to 1,6)	0,05	16,2 (0,90)	16,2 (0,92)
	TAU	18,5 (0,99)	16,0 (1,03)				
Pain acceptance total (CPAQ-R)	ACT	56,2 (1,10)	61,3 (1,21)	−5,2 (−8,3 to −2,1)***	0,32	63,5 (1,29)	62,1 (1,29)
	TAU	54,8 (1,38)	56,1 (1,46)				
Pain willingness (CPAQ-R)	ACT	23,1 (0,64)	25,1 (0,70)	−2,4 (−4,2 to −0,7)**	0,30	26,1 (0,74)	26,1 (0,75)
	TAU	21,9 (0,79)	22,6 (0,84)				
Activity engagement (CPAQ-R)	ACT	32,8 (0,72)	35,8 (0,79)	−2,7 (−4,8 to −0,6) *	0,23	37,0 (0,84)	35,6 (0,84)
	TAU	32,7 (0,91)	33,1 (0,96)				

Note: SF-36v2 = Short Form survey 36 version 2; NRS = Numeric Rating Scale, Pain Intensity is the mean value of strongest, weakest, mean and present pain intensity. HSCL-25 = Hopkins Symptom Check List-25; PCS = Pain Catastrophizing Scale; CPAQ = Chronic Pain Acceptance Questionnaire - Revised; SEM = Standard Error of the Mean. *P < 0,011; **P < 0,007; ***P < 0,001. *^$^ Modified Cohen’s d = Mean difference between groups divided by the standard deviation at baseline.*

**Table 4.** Proportions reporting different responses on Pain intensity, Quality of life and Pain Acceptance after 3-month ACT.

		No improvement	<25% improvement	≥25% improvement
	*N*	*n (%)*	*n (%)*	*n (%)*
Pain intensity*	68	34 (50)	22(32)	12(18)
Quality of life domains**				
Physical Functioning	70	39 (56)	25(36)	6(8)
Role-Physical	70	32 (46)	22(31)	16(23)
Bodily Pain	69	43 (62)	16(23)	10(15)
General Health	70	40 (57)	19(27)	11(16)
Vitality	68	39 (57)	16(24)	13(19)
Social Functioning	67	33 (49)	13(19)	21(32)
Role-Emotional	68	37 (54)	17(25)	14(21)
Mental Health	68	41 (60)	20(30)	7(10)
Pain acceptance total***	70	21 (30)	35(50)	14(20)
Pain willingness ***	70	29 (41)	23(33)	18(26)
Activity engagement ***	70	25 (36)	29(41)	16(23)

Note: ACT = Acceptance and Commitment Therapy at a multidisciplinary pain clinic; *The cutoff levels of Pain intensity were based on a mean of the strongest, weakest, average and present pain intensity assessed by a Numeric Rating Scale; **The cut-off levels of Quality of Life domain were based on scores derived from the Short Form survey 36 version 2; ***Cut-off levels of Pain acceptance scores were based on scores from the Chronic Pain Acceptance Questionnaire - Revised.

#### Secondary outcomes

For the secondary outcome measure of pain acceptance and the subscores pain willingness and activity engagement, we detected statistically significant group differences in favor of ACT compared with TAU with small-to-medium effect sizes (Table 3), and 70% of the patients experienced improved pain acceptance (Table 4).

Given the cross-over design, we had no 6- or 12-month follow-up data on the control group. However, the treatment effect remained at the 6- and 12-month follow-ups with a trend toward improvements. No significant group differences were observed in pain catastrophizing, psychological distress, or fatigue.

All analyses were conducted on the intention-to-treat (ITT) sample. Due to the number of dropouts, we conducted secondary analyses based on per-protocol (PP), but the group comparisons of the primary outcome measures remained nonsignificant.

No adverse events were reported.

## Discussion

To the best of our knowledge, this is the first RCT of a clustered, group-based ACT for individuals suffering from chronic pain. Our main hypotheses were not supported, as no significant between-group differences emerged for the two primary outcome measures pain intensity and health-related quality of life. However, we observed a significant improvement in pain acceptance in favor of ACT compared to TAU provided by the primary health care service, and this was evident for the CPAQ-R sum score as for the subscores pain willingness and activity engagement, representing key concepts within the framework of psychological flexibility, the main target for ACT.^20^

When corresponding studies have demonstrated improved pain acceptance, these studies have typically applied weekly face-to-face sessions or internet-based sessions.^25,67,68^ Our findings extend the evidence by demonstrating that ACT, when clustered into a series of 3-day bouts, may also improve pain acceptance. Our follow-up data further indicate that the effect remains over time, although we cannot conclude as we, due to the semi-crossover design, have no follow-up data for the control (TAU) condition.

Numerous studies^25,69,70^ have shown that psychological flexibility is closely linked to reductions in pain-related symptoms, including decreased pain interference, enhanced well-being, reduced depression, and lower pain-related anxiety.^27,71^ Trompetter et al. (2015) further demonstrated in a clinical trial of an Internet-based ACT intervention for chronic pain that its effects on pain interference and mental distress were mediated by both psychological flexibility and pain catastrophizing.^72^ Similarly, in a cross-sectional study (manuscript in preparation), we have found that pain catastrophizing fully mediates the link between pain acceptance and outcomes like mental distress and physical activity. These findings highlight the crucial role of cognitive and emotional processes in managing pain-related interference and distress.

Although ACT does not directly aim to reduce pain intensity, psychosocial challenges, or physical disability, an indirect effect was anticipated via improved pain acceptance. However, in our trial increased pain acceptance did not result in significant symptom relief across key outcome measures, including pain catastrophizing. Although pain catastrophizing after the ACT intervention did not show significant improvement over TAU, it cannot be ruled out as a potential mediator for pain acceptance.^73^

There are several potential explanations for this lack of symptom relief. The effect size of pain acceptance and the subscores pain willingness and activity engagement were generally small compared to those reported from previous studies.^74^ Thus, the effects may have been below a potential threshold for pain acceptance to act as a process variable affecting pain-related outcome measures.

For ACT to be effective, it necessitates active engagement and sustained commitment from participants.^19,75^ The intensive format of 4.5 to 5.5 hours per day across three consecutive days may have been taxing, potentially limiting participants’ engagement and their ability to fully absorb and retain the therapeutic material.

The intervention design of this trial also differed from other ACT protocols in terms of duration between the bouts. ACT protocols typically emphasize consistent homework and the integration of core principles into daily life–key components for reinforcing therapeutic outcomes. Our design aimed to support participant recovery following travel and the clustered intervention. Still, the 4-week interval between sessions posed a risk to protocol adherence by limiting ongoing support. To address this, we incorporated an interim telephone call from an ACT therapist to provide support, sustain motivation, and promote adherence between the bouts. However, this might not have been sufficient to maintain consistent engagement with the program.

The participants in the current trial described significant fatigue in line with previous findings among patients with chronic pain^76,77^ (Table 2). The intensive intervention format may therefore have been perceived as too exhausting by some, and this might have contributed to the drop-out rate during the intervention.

The number of participants who withdrew before the intervention is a point of concern in this study. Figure 1 (Flow chart) shows the attrition rates at various stages of the study. The extended delay between randomization and the initiation of the intervention, sometimes several months, may have contributed to these withdrawals. This may indeed have reduced their motivation to participate and introduced conflicts with other responsibilities or priorities. Participants both in the “early” and “late” ACT condition did experience a delay due to the inclusion practice and the timing of the ACT intervention, and this might explain why attrition was observed relatively alike in the “early” and “late” ACT conditions. The trial was partly run during the COVID pandemic, and this extended the delay and probably discouraged participation.

As indicated in Table 4, approximately 50% of participants reported improvement. This heterogeneity within the sample indicates different effects across various subgroups and attenuates the overall treatment effect, thereby limiting the capacity to detect statistically significant differences between groups. Although baseline levels of the outcome measures were consistent with those observed in similar studies (e.g., Trompetter et al., Wetherell et al., and Vasilou et al.), further analyses to identify individual characteristics that distinguish responders from nonresponders could offer valuable insights into the factors influencing treatment efficacy and provide ways for tailoring interventions to specific subgroups within the chronic pain population. However, conducting such detailed analyses would require a larger sample size than was available in the current trial.

As part of the main research project, we have conducted qualitative interviews with a subset of participants to gain a deeper understanding of their experiences with the ACT intervention, including their motivations and perceived barriers to participation. These findings will be reported in a separate publication.

### Strength and limitations

Our trial has strengths as well as noteworthy limitations. The randomized controlled and prospective design with several measurements over time is the preferred method for establishing evidence for the effects of an intervention. The treatment intervention was, moreover, conducted within an established university hospital clinic, with participants referred for chronic pain issues. This setting enhances the external validity of our findings.

The therapists had various professional backgrounds, and this may have introduced variability in how the treatment was delivered. To strengthen treatment integrity and fidelity, we therefore selected clinically experienced therapists, well-trained in the ACT procedures with monthly supervision throughout the study period. Furthermore, the therapists worked in pairs throughout the treatment sessions. Even so, additional efforts such as video-recording the therapy sessions and rating therapists’ adherence and fidelity to the treatment manual would have further ensured and strengthened the treatment integrity of the intervention.

Another factor relates to the participants’ adherence to assigned homework and their commitment to incorporate ACT principles into daily life, as limited adherence may undermine the intervention’s efficacy. Regrettably, we lack data on participants’ actual engagement and adherence levels within the intervention. Self-reported data on homework completion and practice of techniques between the treatment sessions could have offered valuable insights into the adherence.

As in comparable studies, our results are exclusively based on self-report questionnaires. Although the selected outcome measures have shown good validity and reliability, self-reported data is associated with response bias and limited memory recall. Another potential contributing factor might be related to the assessment of psychological flexibility. We utilized the CPAQ-R questionnaire, which primarily focuses on pain acceptance, particularly addressing the aspects activity engagement (the pursuit of life activities regardless of pain), and pain willingness (recognizing the limitations of avoidance and control). This tool primarily measures the psychological processes: acceptance, committed action, and values, and does not encompass the full range of therapeutic psychological processes within ACT that may contribute to increased psychological flexibility. Including measures of other processes such as cognitive defusion (detaching from unhelpful thoughts about pain), present moment awareness (mindfulness practices), and self-as-context (flexible perspective of self beyond pain-related identity) might have provided valuable information and perhaps detected effects of the intervention not captured by the present study.

With a semi-crossover design, this trial includes no control data beyond 3 months. We can therefore not conclude on long-term efficacy even though pain acceptance remained stable within 12 months.

## Conclusion

This trial investigated the efficacy of a clustered, group-based ACT intervention for chronic pain, delivered in a series of 3-day bouts. Pain acceptance processes, such as pain willingness and activity engagement were enhanced. However, the primary hypothesis was not supported, as no significant effects were observed on pain intensity or health-related quality of life.

These findings provide new evidence for this approach and highlight the need for further exploration, including qualitative studies, to better understand participants’ experiences and the mechanisms driving these outcomes.

## Supplementary Material

2025_Article_second_revison_ACT_RCT_Track changes.docx
